# 

**Published:** 1875-08

**Authors:** 


CONTENTS.

PAGE.
Diseases of Summer Time ....235
Summer Complaint of Children ....236
Tyranny Over Women ....237
Graham Bread ....238
Our Summer at Maplewood—Chapter vii.—At Dinner ...239
Hearty Breakfasts ....245
Save the Forests ....246
A Great Book ....247
Texas Beef ....248
Restaurants ....248
A Broken Heart ....249

PAGE.
A Gentleman in Disguise ....250
Cutaneous Eruptions ....250
Where Shall we Spend the Summer? ....251
Soap Which Heals ....255
Cotton Felt ....256
Poisoning by Plants ....257
Zero Market Basket ....258
Bilious ....259
For Services Rendered ....260
Amateur Printing ....260
Anatomy in Schools ....261
Dress Reform ....263

